# Anticipated help seeking behaviour and barriers to seeking care for possible breast and cervical cancer symptoms in Uganda and South Africa

**DOI:** 10.3332/ecancer.2021.1171

**Published:** 2021-01-14

**Authors:** Jennifer Moodley, Deborah Constant, Amos Deogratius Mwaka, Suzanne Emilie Scott, Fiona Mary Walter

**Affiliations:** 1 Women’s Health Research Unit, School of Public Health and Family Medicine, Faculty of Health Sciences, University of Cape Town, Observatory, Cape Town, 7925, South Africa; 2 Cancer Research Initiative, Faculty of Health Sciences, University of Cape Town, Observatory, Cape Town, 7925, South Africa; 3 South African Medical Research Council Gynaecology Cancer Research Centre, Faculty of Health Sciences, University of Cape Town, Observatory, Cape Town, 7925, South Africa; 4 Department of Medicine, School of Medicine, College of Health Sciences, Makerere University, Upper Mulago Jill Road PO Box 7072, Kampala, +256, Uganda; 5 Centre for Oral, Clinical and Translational Sciences, Faculty of Dentistry, Oral and Craniofacial Sciences, King’s College London, SE1 9RT, UK; 6 The Primary Care Unit, Department of Public Health and Primary Care, University of Cambridge, CB1 8RN, UK; a https://orcid.org/0000-0002-9398-5202; b https://orcid.org/0000-0002-7176-9963; c https://orcid.org/0000-0001-7952-2327; d https://orcid.org/0000-0001-5536-9612; e https://orcid.org/0000-0002-7191-6476

**Keywords:** help-seeking, breast cancer, cervical cancer, barriers to care, symptoms

## Abstract

**Objectives:**

Breast and cervical cancers are leading causes of cancer morbidity and mortality in sub-Saharan Africa. Most women present with advanced-stage disease and have poor outcomes. This study aimed to describe anticipated help-seeking behaviour for possible breast and cervical cancer symptoms, barriers to accessing health care and factors associated with less timely anticipated help-seeking in urban and rural settings in Uganda and South Africa (SA).

**Methods:**

We conducted a cross-sectional community-based survey between August and December 2018. Data were collected from one randomly selected woman per household using the African Women Awareness of CANcer breast and cervical cancer tool. Anticipated help-seeking behaviour was dichotomised into waiting <1week or ≥1 week to seek care. Multivariable analysis identified factors associated with anticipated help-seeking behaviour.

**Results:**

One thousand, seven hundred fifty-eight women participated (Uganda 873, SA 885, median age 34, interquartile ranges 26–47). Most would discuss symptoms with someone close to them (87.7% for breast, 83.0% for cervical symptoms). The majority anticipated seeking care from a health facility in <1 week: 86.1% and 88.0%, respectively, for breast and cervical symptoms. 38.7% of women expected to encounter at least 1 barrier when seeking care. Lack of money for transport or clinic costs was the most common barrier (24.6% of participants). For both cancers and in both countries, women who reported more barriers were significantly less likely to anticipate seeking timely care. In SA, rural location was also associated with longer anticipated time to seek care, adjusted prevalence ratio (aPR) 2.92, 95% confidence interval (CI) 1.48–5.76 and aPR 2.42, 95% CI 1.08–5.45 for breast and cervical cancer, respectively.

**Conclusion:**

Interventions that improve community level cancer knowledge and highlight the importance of prompt help-seeking for possible symptoms are important to promote timely care seeking. In addition, addressing financial barriers by reducing transport and clinic costs and tackling geographical inequities in access to care could support women in seeking timely care for possible symptoms.

## Introduction

The global burden of cancer is increasing[1]. By 2030, an estimated 24 million people worldwide will develop cancer and 13 million people will die annually from cancer, with 75% of deaths in low- and middle-income countries (LMICs) [2, 3]. The increasing cancer burden in LMICs is attributed to the high prevalence of risk factors, ageing societies and inequalities in care-seeking, provision and quality of care [4].

Breast and cervical cancers are leading causes of cancer morbidity and mortality among women in LMICs [3]. Cervical cancer accounts for 16% of the total cancer burden in LMICs, with Africa having the highest age standardised incidence rate (ASIR), 27.6 per 100,000, compared to the world ASIR of 13.1 per 100,000 [3]. Whilst cervical cancer is the leading cause of cancer deaths among women in Africa, breast cancer is the most commonly diagnosed cancer [3]. Although breast cancer incidence rates in Africa are lower than those reported by high-income countries (HICs), mortality rates are similar [3]. For example, ASIRs for breast cancer in UK, South Africa (SA) and Uganda are 94, 49 and 21 per 100,000 women, respectively, whilst age-standardised mortality rates are 14, 16 and 10 per 100,000 women, respectively [3].

Stage at diagnosis is an important predictor of cancer outcomes, with advanced stage cancer (stages III or IV) being least responsive to curative treatment. Most African countries do not have cervical or breast cancer screening programmes and the majority of cancers are diagnosed symptomatically and at an advanced stage [4]. In sub-Saharan Africa (SSA), 64% of breast and 66% cervical cancers are diagnosed at an advanced stage [5, 6]. By comparison, fewer than a quarter of breast and cervical cancers (21% and 25%, respectively) are diagnosed at an advanced stage in the UK [7]. This contributes to the marked differences in age standardised relative survival (ASRS) between SSA and HICs. Between 2008 and 2015, the 5-year breast cancer survival rate in SSA was 66% which is similar to the survival rates observed 60 years ago in HICs [5]. Current cervical cancer survival rates in SSA are almost half that seen in the UK (5-year ASRS in SSA 33% compared to 67% in UK) [6].

A key goal of comprehensive cancer control is a shift to earlier stage at diagnosis [4]. Minimising the time to diagnosis is dependent on knowledge of relevant symptoms and risk factors; appropriate appraisal of possible cancer symptoms; timely presentation to primary healthcare providers; appropriate assessment at the primary healthcare level and timely access to referral and treatment centres [8, 9]. The current research focuses on the earlier parts of the diagnostic pathway—the decision to seek help. Understanding factors that impact on whether and when symptomatic women seek care can inform efforts to promote earlier diagnosis of cancer, and ultimately better cancer survival. Research in SSA has shown that personal (e.g. knowledge of symptoms, requiring spousal/partner permission to attend health care services), disease (e.g. disease site, symptoms and progression) and healthcare factors (e.g. access to primary health care facilities, facility waiting times) are associated with the timeliness of seeking care [10–13]. However, most of this understanding comes from research undertaken with patients already diagnosed with cancer using small samples and a retrospective design. This has the potential for recall bias, particularly for those who were diagnosed a long time after first noticing symptoms. Little is known about anticipated behaviour prior to a cancer diagnosis, particularly in SSA. Information on anticipated help-seeking behaviour is important to enable the development of effective interventions to reduce delay and promote timeliness of presentation and diagnosis. The aim of this study was to describe women’s anticipated help-seeking behaviour for possible breast and cervical cancer symptoms, to describe perceived barriers to accessing health care, and to determine factors associated with less timely anticipated help-seeking in urban and rural settings in Uganda (a low-income country) and SA (a middle-income country).

## Methods

### Study design

We conducted a community-based cross-sectional survey of women aged 18 years and over in one urban and one rural site in both Uganda and SA (total of four sites). Information on the study sites, sample size, data collection methods and results related to awareness of breast and cervical cancer risk factors, symptoms and lay beliefs have been published elsewhere [10] and are only described briefly here. Methods relevant to the help-seeking behaviour and barriers to care are described in more detail below.

### Participants

At each site, households were selected using systematic random sampling. One woman per identified household was then randomly selected and invited to participate in the study. Women unable to speak either English, Acholi (Uganda) or isiXhosa (SA), and women with a history of breast or cervical cancer, were excluded.

### Data collection

Data were collected by trained interviewers using a hand-held electronic tablet customised with the African Women Awareness of CANcer (AWACAN) tool (Moodley *et al* AWACAN validation), which is available in English, Acholi and isiXhosa. Women were interviewed face-to-face in their homes, either in their local language (Acholi in Uganda and isiXhosa in SA) or in English depending on their preference.

### Measures of socio-demographic variables

We collected the following socio-demographic details: age, relationship status, educational level, employment status and information on access to assets. We constructed a composite asset index to describe socioeconomic status using principal component analysis on a model that included asset variables with a positive correlation at *p* ≤ 0.01. The asset index was then divided into terciles.

### Measures of cancer awareness

#### Risk factor and symptom awareness

Risk factor and symptom awareness for both cancers were measured using a closed/prompted question format as laid out in the AWACAN tool: 13 items on breast cancer risk factors, 15 items on breast cancer symptoms and 11 items each on cervical cancer risk factors and symptoms. In addition, the AWACAN tool includes prompted lay belief items: six on breast cancer risk factors and four on cervical cancer risk factors. Lay beliefs refer to locally held beliefs for which there is no current scientific evidence and play an important role in risk interpretation and help-seeking behaviour [10, 11]. Women scored ‘1’ for ‘Yes’ and ‘0’ for ‘No’ or ‘Don’t know’. Scores for each item were summed to form separate knowledge scores for breast cancer risk factors (0–13), breast cancer symptoms (0–15), breast cancer lay beliefs (0–6), cervical cancer risk factors (0–11), cervical cancer symptoms (0–11) and cervical cancer lay beliefs (0–4). Higher scores indicated better recognition of symptoms and risk factors and greater endorsement of lay beliefs.

#### Confidence in symptom detection

Confidence in symptom detection was measured by asking respondents ‘Are you confident that you would notice a change in your breasts/ symptom that could be cervical cancer ?’ (Yes = 1, No = 0).

### Measure of barriers to seeking care

To measure barriers, the AWACAN tool stem question asks ‘Would any of the following reasons make it difficult for you to see the nurse or clinical officer or doctor if you noticed a symptom or sign which you think may be serious, for example, a change in your breast or a change in the mouth of your womb or cervix that could be cancer?’.[14]. Barriers to seeking care included five personal emotional barriers (e.g. ‘worried about what they may find wrong’), four health service barriers (e.g. ‘long waiting times at health facility’) and three practical barriers (e.g. ‘lack of money for transport or facility charges’). Responses were recorded as Agree, Disagree or Don’t know. The overall barrier score was the number of ‘Agree’ responses (range 0–12).

### Measures of anticipated help-seeking

The AWACAN tool has questions relating to anticipated help-seeking behaviours for each cancer separately. These include whether the participant would try self-medication, tell someone close to them, visit a traditional healer and visit a health care facility. For each of these items, participants could respond Yes, No or Don’t Know. Anticipated time to seeking care at a health facility was recorded in pre-determined categories: never; <1 week; ≥1 week <1 month; ≥1 month <3 months; ≥ months. Responses were then dichotomised into <1 week (shorter) versus ≥1 week (longer) to seek care.

### Statistical analysis

We analysed the data using Stata version 15, using valid percentages to report sociodemographic data and anticipated help-seeking responses. Medians and interquartile ranges (IQR) are reported for continuous variables. As Ugandan and South African sociodemographic indicators showed significant differences, we did the bivariate and multivariable analysis by country to best demonstrate these context specific effects. As longer time to seek help (≥1 week) occurred in >10% for both cancers in each country, we performed Poisson regression with robust variance to identify factors associated with anticipated time to seek help, individually and independently in a multivariable model. Adjusted prevalence ratios (aPR) and 95% confidence intervals (CIs) are reported. The multivariable Poisson regression models included the following variables: location, age, relationship status, education level, paid work and asset index, risk factor and symptom knowledge scores, risk factor lay beliefs, barriers and confidence in detecting a symptom. Statistical significance was set at the *p* < 0.05 level.

### Ethics

Ethics approval for the study was obtained from University of Cape Town, Faculty of Health Sciences Human Research Ethics Committee (HREC 544/2016), the Lacor Hospital Institutional Research Ethics Committee (LHIREC 027/11/2016) and the Ugandan National Council of Science and Technology (HS60ES). Written informed consent was obtained from all participants.

### Public involvement

At the start of the project, community-based project advisory committees (PACs) were set up in each country. The PACs included local government councillors, traditional leaders as well as representatives from the cancer registry and public sector health services (health service providers and managers). The AWACAN tool used in the survey was developed with input from PAC members. In this study, PAC members assisted in gaining access to communities, recruiting local women as field workers in each site and providing guidance on field worker safety. AWACAN results have been discussed with PAC members and field workers in a series of meetings.

## Results

Between August and December 2018, a total of 1,941 households were visited in Uganda and SA, and 1,758 participants recruited. The overall refusal rate was 6.3%. Details of site recruitment, reasons for refusal and ineligibility and final enrolment numbers have been published previously [10].

### Participant profile, cancer awareness and confidence in detecting symptoms

Participant characteristics by country and site are outlined in Table 1. The overall median age of respondents was 34 years (IQR 26–47). For each country, there were significant urban/rural differences in participants age, relationship status, educational level, work status and asset index profile. A higher proportion of women in rural Uganda were married and had no or incomplete primary schooling compared to other sites. Overall, 66.3% of participants did no paid work, with the highest rate in rural SA (91.8%). Results on awareness of breast and cervical cancer symptoms, risk factors and lay beliefs are described elsewhere [10] and summary results included in Table 1. Most women (88.3%) were confident that they would notice a breast change. Overall 66.5% of women reported that they were confident in noticing cervical symptoms, with differences in confidence levels between countries and between urban and rural settings.

### Barriers to seeking medical help

Overall, 38.7% of women reported at least one barrier to seeking medical care: 15.3% experienced at least one emotional barrier, 22.7% at least one health service barrier and 27.5% at least one practical barrier. Table 2 provides information on the emotional, practical and health system barriers to accessing care for possible breast and cervical cancer symptoms by each site. Overall the most common barrier reported was financial, i.e. not having money for transport or clinic costs, which was reported by 24.6% of all respondents. In SA, significantly more rural compared to urban women experienced a financial barrier (18.7% versus 6.5%. *p* < 0.01). In Uganda, a high and similar proportion of rural women and urban women reported a financial barrier (38.2% and 36.2%, respectively, *p* = 0.35).

### Anticipated response to possible breast and cervical cancer symptoms

Most women reported that they would discuss possible symptoms with someone close to them (87.7% for breast, 83.0% for cervical symptoms), with significant differences between urban and rural participants in SA (see Table 3). A quarter of women would try self-medication for breast symptoms and a fifth for cervical symptoms, with rural women significantly more likely to try self-medication for both cancers compared to urban women. Few women reported that they would visit a traditional healer (9.3% for breast and 8.1% for cervical—Table 3 provides details by each site). Of those that would visit a traditional healer for a symptom, just over half would do this in less than 1 week.

The majority of women anticipated that they would visit a healthcare facility within 1 week of noticing a breast symptom (86.1%) or cervical symptom (88.0%) (Table 3).

### Factors associated with longer anticipated time to seek healthcare

#### (a) for possible symptoms of breast cancer

Associations between independent factors and longer anticipated help-seeking for possible symptoms of breast cancer are presented in Table 4. Multivariable analyses indicated that in SA, significant determinants of longer anticipated time to seek healthcare were rural compared to urban location (aPR 2.92, 95% CI 1.48–5.76), experiencing more barriers (aPR 1.18, 95% CI 1.08–1.29), lower risk factor knowledge (aPR 0.91, 95% CI 0.83–0.99) and having more assets (aPR 0.27, 95% CI 0.13–0.55, aPR 0.37, 95% CI 0.17–0.79 for middle and lower terciles compared to upper tercile, respectively). In Uganda, factors significantly associated with longer anticipated time to seek healthcare were higher risk factor knowledge (aPR 1.07, 95% CI 1.01–1.13), experiencing more barriers to care (aPR 1.33, 95% CI 1.26–1.40) and endorsing fewer lay beliefs (aPR 0.87, 95% CI 0.78–0.97).

#### (b) for possible symptoms of cervical cancer

Factors associated with longer anticipated help-seeking for possible symptoms of cervical cancer are presented in Table 5. As with possible breast cancer symptoms, the multivariable analyses indicated that in SA, women living in rural areas, those experiencing more barriers and those with more assets (aPR 0.34, 95% CI 0.15–0.80) were more likely to indicate they would wait longer before seeking help (aPR 2.42, 95% CI 1.08–5.45 and aPR 1.19, 95% CI 1.06–1.34, respectively). Among Ugandan women, experiencing more barriers to accessing care was significantly associated with less timely anticipated help-seeking (aPR 1.23, 95% CI 1.16–1.30).

## Discussion

Prompt help-seeking when a possible symptom is discovered is an important step towards timely cancer diagnosis [4]. This is the first community-based study in SSA reporting on anticipated help-seeking behaviour and barriers to care for possible breast and cervical cancer symptoms. We found that most women did not anticipate delay in seeking care—86% and 88% would seek care at a health facility within 1 week for a possible breast or cervical symptom, respectively. This contrasts starkly with actual patient intervals (time between noticing a symptom and presenting to a health facility) reported in LMIC settings [11, 15–17]. For example, for breast cancer, Rayne *et al* [15] and Moodley *et al* [17] reported that only 33% and 25%, respectively, of breast cancer patients attended a health facility within 1 week of noticing a symptom. This discrepancy between hypothetical and actual help-seeking has also been reported in HIC settings [18]. A review of studies measuring time to seeking care for a number of different cancers found that most people anticipated seeking care within 1 week, whereas most patients actually waited for more than 2 months before seeking medical care [18]. This is likely due to the bulk of delay in seeking help arising in the appraisal interval, whereby symptoms are misinterpreted as minor, manageable conditions that do not require attention from a healthcare professional [19]. In this study, we studied the help-seeking interval [8, 9] and have demonstrated that even when women do consider cancer (or something serious) a proportion of women would still not seek help straightway and a number of barriers to healthcare use are key in explaining this delay. Thus, whilst improving cancer knowledge is important, our findings suggest that to improve timely diagnosis, interventions must be directed at more than just improving symptom knowledge and include support to overcome emotional, health service and practical barriers to seeking help.

In our study, almost 40% of women expected to encounter at least one barrier when seeking care for a possible breast or cervical cancer symptom. Importantly, women who reported more barriers were significantly less likely to seek timely care in both Uganda and SA. Both the Alma-Ata Declaration of 1978 and the Astana Declaration of 2018 identify access to basic health care as a cornerstone to achieving health for all [20, 21]. In pursuit of this, many countries have committed to strengthening primary health care. Yet for many people, access to care for possible cancer symptoms remains a challenging process of negotiating a series of barriers [22–26]. Identifying these barriers is a key first step that can inform ways to mitigate their impact and foster timely presentation to health services.

As in other LMIC contexts, transportation and facility costs emerged as key access barriers in our study, especially for rural residents [23, 26, 27]. Research has shown that experiencing financial difficulty delays symptomatic presentation and is associated with advanced stage at cancer diagnosis [11, 18, 28]. In countries such as SA where user fees for primary health services have been abolished, there are still transport-related costs borne by individuals. This is a particular burden where health facilities are often far from people’s homes. Our finding of a strong association between rural location and anticipated delay in seeking care in SA supports the findings that despite government efforts to make health care more accessible by expanding the number of health facilities, geography remains an important barrier to equitable access to health care [27, 29]. Addressing financial and geographic access barriers is complex and will require a cross-sectoral approach.

Facility waiting times are another important dimension of access to health care [30] and long waiting time have been reported and contribute to patient dissatisfaction [22, 23, 31, 32]. Relatively few of our participants (13.3%) felt that long health facility waiting times would be a barrier to seek care for a possible breast or cervical cancer symptom. It is possible that waiting times differ by levels of health care. Our study enquired about accessing primary health care facilities, whereas the other studies [22, 31, 32] were conducted at secondary and tertiary facilities. Research measuring the facility waiting times at the various levels of health care (primary, secondary and tertiary) in LMIC settings will be important in identifying specific bottlenecks in the pathway to cancer care and serve as points for future interventions to improve timely access to care.

We found important differences in the types of barriers reported in our LMIC settings compared to studies conducted in HIC settings [25, 26, 33]. For example, in the UK, concern about wasting a provider’s time has been reported as an important barrier to accessing care (30% Niksic *et al* [26], 19.5 % Moffat *et al* [25]), whereas only 3.2% of women in our study felt this would be a barrier. In a study on anticipated help-seeking for cancer symptoms among ethnic minority groups in the UK, Waller *et al* [33] found that for more than a third of participants felt that being too embarrassed would be a reason to delay seeking care [33]. In contrast, less than 10% of our participants felt that this would be a barrier to seeking care. These difference highlights the importance of local studies in informing intervention development.

Most women in our study reported that they would discuss a symptom they considered serious with a family member or friend. This underscores the importance of community level interventions that support women to seek care. Traditional healers are an important part of the health system in Africa [11, 13, 28, 34]. We found that few women (<10%) anticipated seeking care from a traditional healer for a possible breast or cervical cancer symptom. This low anticipated use could reflect a decline in use of traditional healers, reluctance to disclose use of traditional medicine or that traditional healers might only be visited later in the care pathway. Further qualitative studies could shed light on this.

We found no association between socio-economic status as measured by the asset index and anticipated delay in Uganda, but in SA women with more assets were more likely to delay in seeking care for possible symptoms. Although most studies have found that lower socio-economic status is associated with greater patient delay in seeking care [11, 35], some studies have either reported no association or greater delays in higher compared to lower socio-economic groups [16, 36, 37]. For example, Poum *et al* [37] found that patient delay for breast cancer symptoms was significantly associated with higher family income [37]. These findings highlight the importance of interventions targeting all income groups. In addition, further research on why SA women with more assets are likely to delay seeking care will be helpful in designing interventions to promote timely care seeking.

Similar to other international studies, we found that greater breast cancer risk factor knowledge was associated with more timely help-seeking behaviour in SA [33, 35]. However, in Uganda, women with higher breast cancer risk factor knowledge were more likely to delay seeking care. Interestingly, in Uganda, endorsement of lay beliefs was associated with more timely help-seeking behaviour. Further qualitative research could shed light on the interplay between risk factor knowledge, endorsement of lay beliefs and anticipated delay in seeking care.﻿

A key strength of our study was that data collection interviews were conducted in the participants home language using a validated questionnaire [14]. Our study also had limitations, as it was hypothetical in nature and responses to a hypothetical symptom may not translate into real-life behaviour. A prospective study design in which help-seeking behaviour for symptoms is investigated as it occurs could be more insightful and less biased. However, prospective designs can have drawbacks, such as the large sample size and resources required for such a study [38]. In our study, selection of sites was directed at bringing forth the uniqueness of each context. While our study offers distinctive SSA perspectives, the four sites are not representative at a country level. Any interpretation and use of results should take this into consideration. We acknowledge that our study focused on barriers and anticipated care-seeking when a symptom is identified as being serious; however, delay in seeking care could also be due to not recognising a symptom.

## Conclusion

We identified factors affecting anticipated help-seeking for possible breast and cervical cancer symptoms. Interventions that improve community level cancer knowledge and highlight the importance of prompt help-seeking for possible symptoms are important to promote timely care seeking. In addition, addressing financial barriers by reducing transport and clinic costs and tackling geographical inequities in access to care could support women in seeking timely care for possible symptoms.

## Conflicts of interest

The authors declare that they have no conflict of interest.

## Funding statement

Research reported in this article was jointly supported by the Cancer Association of South Africa, the University of Cape Town and the SA Medical Research Council with funds received from the SA National Department of Health, GlaxoSmithKline Africa Non-Communicable Disease Open Lab (via a supporting grant Project Number: 023), the UK Medical Research Council (via the Newton Fund). Authors retained control of the final content of the publication. The funders had no role in study design, data collection and analysis, decision to publish or preparation of the manuscript.

## Authors’ contributions

JM, FMW, SES and ADM conceptualised and designed the study.

JM, ADM and DC oversaw data collection.

DC curated the data.

DC and JM conducted the quantitative analysis and all authors reviewed the study results.

JM prepared the first draft, incorporated revisions and prepared the final draft. All the authors reviewed drafts and approved the final manuscript.

## Figures and Tables

**Table 1.** Participant profile.

	SA			Uganda			Total
	Urban	Rural	*p*-value^a^	Urban	Rural	*p*-value^a^	
	*n* (%) *n* = 445	*n* (%) *n* = 428		*n* (%) *n* = 458	*n* (%) *n* = 427		*n* (%) *n* = 1758
**Age (years)**							
18–29	130 (30.4)	106 (25.2)	<0.001	216 (47.2)	186 (44.0)	0.04	638 (36.9)
30–49	221 (51.6)	170 (40.4)		174 (38.0)	146 (34.5)		711 (41.1)
≥50	77 (18.0)	145 (34.4)		68 (14.9)	91 (21.5)		381 (22.0)
Median (IQR)	36 (28–45)	40 (29–54)		30 (24–40)	32 (23–46)		34 (26–47)
**Relationship status**							
Married/living with a partner	185 (41.8)	200 (46.7)	0.01	300 (65.5)	314 (73.5)	0.01	999 (56.9)
No partner/not living with partner	224 (50.6)	174 (40.7)		36 (7.9)	17 (4.0)		451 (25.7)
Separated/divorced/widowed	34 (7.7)	54 (12.6)		122 (26.6)	96 (22.5)		306 (17.4)
**Highest educational level**							
No schooling and primary incomplete	22 (5.0)	162 (38.0)	<0.001	171 (37.3)	328 (76.8)	<0.001	683 (39.0)
Primary complete and secondary incomplete	196 (44.3)	178 (41.8)		167 (36.5)	84 (19.7)		625 (35.6)
Secondary complete or more	224 (50.7)	86 (20.2)		120 (26.2)	15 (3.5)		445 (25.4)
**Paid work**							
Yes	226 (51.6)	35 (8.2)	<0.001	217 (47.6)	110 (25.8)	<0.001	588 (33.7)
No	212 (48.4)	391 (91.8)		239 (52.4)	317 (74.2)		1159 (66.3)
**Asset index**							
Upper tercile	326 (73.3)	6 (1.4)	<0.001	253 (55.2)	70 (16.4)	<0.001	655 (37.3)
Middle tercile	79 (17.8)	182 (42.5)		122 (26.6)	210 (49.2)		593 (33.7)
Lower tercile	40 (9.0)	240 (56.1)		83 (18.1)	147 (34.4)		510 (29.0)
**Breast cancer awareness**							
Median (IQR) risk factor awareness score	5 (3–9)	4 (2–6)	<0.001	5 (2–7)	5 (3–7)	0.38	4 (3–7)
Median (IQR) symptom awareness score	15 (14–15)	13 (10–14)	<0.001	13 (11–15)	13 (10–15)	0.24	14 (11–15)
Median (IQR) number of lay beliefs endorsed	5 (4–5)	4 (2–5)	<0.001	4 (4–-5)	5 (3–5)	0.82	4 (4–5)
**Cervical cancer awareness**							
Median (IQR) risk factor awareness score	9 (7–10)	8 (6–9)	<0.001	7 (5–9)	8 (6–9)	0.01	8 (6–9)
Median (IQR) symptom awareness score	9 (8–11)	6 (8–10)	<0.001	9 (7–10)	9 (6–10)	0.90	9 (7–10)
Median (IQR) number of lay beliefs endorsed	2 (2–3)	2 (2–3)	<0.001	2 (2–3)	2 (2–3)	0.46	2 (2–3)
**Confident in noticing a breast change**	383 (86.1)	403 (94.2)	<0.001	410 (89.5)	357 (83.6)	0.01	1553 (88.3)
**Confident in noticing a cervical symptom**	371 (83.4)	333 (77.8)	0.04	274 (59.8)	191 (44.7)	<0.001	1169 (66.5)

There were missing data for: Age: SA urban; *n* = 17, Rural; *n* = 7. Uganda rural; *n* = 4. Total; *n* = 28; Relationship status: SA urban; *n* = 2. Total; *n* = 2; Highest educational level: SA urban; *n* = 3, Rural; *n* = 2. Total; *n* = 5; Paid work: SA urban; *n* = 7, Rural; *n* = 2. Uganda urban = 2. Total; *n* = 11

a Chi squared tests for proportions and Wilcoxon Signed Rank Test for medians IQR, Interquartile range

**Table 2.** Barriers to help seeking for breast and cervical cancers.

	SA			Uganda			Total
	**Urban**	**Rural**	***p*-value^b^**	**Urban**	**Rural**	***p*-value^b^**	
	*n* (%) *n* = 445	*n* (%) *n* = 428		*n* (%) *n* = 458	*n* (%) *n* = 427		*n* (%) *n* = 1,758
**Personal emotional barriers**							
Worried about tests that may be done	33 (7.4)	34 (7.9)	0.77	37 (8.1)	24 (5.6)	0.15	128 (7.3)
Worried about what they might find wrong	25 (5.6)	20 (4.7)	0.53	27 (5.9)	30 (7.0)	0.49	102 (5.8)
Feel embarrassed	25 (5.6)	26 (6.1)	0.77	27 (5.9)	33 (7.7)	0.28	111 (6.3)
Not confident to talk about symptoms with the doctor	23 (5.2)	27 (6.3)	0.47	28 (6.1)	37 (8.7)	0.15	115 (6.3)
Worried about wasting doctor’s time	20 (4.5)	21 (4.9)	0.77	6 (1.3)	9 (2.1)	0.36	56 (3.2)
Fatalistic cancer belief—it is a serious and will die anyway^a^	2 (0.5)	12 (2.8)	0.01	17 (3.7)	26 (6.1)	0.10	57 (3.2)
**Personal practical barriers**							
Lack of money for transport or facility charges	29 (6.5)	80 (18.7)	<0.001	161 (35.2)	163 (38.2)	0.35	433 (24.6)
Other priorities	11 (2.5)	20 (4.7)	0.08	25 (5.5)	45 (10.5)	0.01	101 (5.8)
Husband/partner would not allow me to go^a^	7 (1.6)	10 (2.3)	0.41	35 (8.2)	18 (3.9)	0.01	70 (4.0)
**Health system barriers**							
Long health facility waiting times	83 (18.7)	62 (14.5)	0.10	48 (10.5)	41 (9.6)	0.66	234 (13.3)
Past bad experience at health facility	35 (7.9)	27 (6.3)	0.37	47 (10.3)	34 (8.0)	0.24	143 (8.1)
Health practitioner might not understand my language or culture	8 (1.8)	23 (5.4)	0.01	57 (12.5)	52 (12.2)	0.90	140 (8.0)

a *N* = 1,757 (missing data for one woman in rural SA)

b Chi squared tests for proportions and Wilcoxon Signed Rank Test for medians

**Table 3.** Anticipated responses to breast cancer and cervical cancer symptoms.

	SA			Uganda			Total
	Urban	Rural	*p*-value^a^	Urban	Rural	*p*-value^a^	
	*n* (%) *n* = 445	*n* (%) *n* = 428		*n* (%) *n* = 458	*n* (%) *n* = 427		*n* (%) *n* = 1,758
**Response to noticing a breast change**							
Try self-medication	110 (24.7)	157 (36.7)	<0.001	76 (16.6)	106 (24.8)	<0.01	449 (25.5)
Tell someone close to you	359 (80.7)	405 (94.6)	<0.001	395 (86.2)	382 (89.5)	0.14	1,541 (87.7)
Visit the healthcare facility in:							
<1 week	403 (90.6)	378 (88.3)	0.28	380 (83.0)	353 (82.7)	0.91	1,514 (86.1)
1 week or more	42 (9.4)	50 (11.7)		78 (17.0)	74 (17.3)		244 (13.9)
Visit a traditional healer	53 (11.9)	37 (8.64)	0.11	32 (7.0)	41 (9.6)	0.16	163 (9.3)
Time to visit a traditional healer	***n* = 51**	***n* = 35**		***n* = 32**	***n* = 41**		***n* = 159**
<1 week	34 (66.7)	27 (77.1)	0.29	9 (28.1)	17 (41.5)	0.24	87 (54.7)
1 week or more	17 (33.3)	8 (22.9)		23 (71.9)	24 (58.5)		72 (45.3)
**Response to noticing a cervical symptom**							
Try self-medication	90 (20.2)	145 (33.9)	<0.001	62 (13.5)	83 (19.4)	0.02	380 (21.6)
Tell someone close to you	351 (78.9)	403 (94.4)	<0.001	362 (79.0)	342 (80.1)	0.70	1458 (83.0)
Visit the healthcare facility in:							
<1 week	406 (91.2)	384 (89.7)	0.45	403 (88.0)	355 (83.1)	0.04	1548 (88.0)
1 week or more	39 (8.8)	44 (10.3)		55 (12.0)	72 (16.9)		210 (12.0)
Visit a traditional healer	43 (9.7)	34 (8.0)	0.38	28 (6.1)	37 (8.7)	0.15	142 (8.1)
Time to visit a traditional healer	***n* = 43**	***n* = 33**		***n* = 25**	***n* = 37**		***n* = 138**
<1 week	29 (67.4)	22 (6.7)	0.94	10 (40.0)	14 (37.8)	0.86	75 (54.4)
1 week or more	14 (32.6)	11 (33.3)		15 (60.0)	23 (62.2)		63 (45.7)

There were missing data for Time to visit a traditional healer: SA rural *n* = 1; Uganda urban *n* = 3

a Chi squared tests for proportions and Wilcoxon Signed Rank Test for medians

**Table 4.** Factors associated with longer anticipated time to seek care for possible symptoms of breast cancer.

	SA				Uganda			
	**Prevalence ratio (PR)**	**95% CI PR**	**Adj PR**	**95% CI Adj PR**	**PR**	**95% CI PR**	**Adj PR**	**95% CI Adj PR**
**Location**								
Urban	Referent		Referent		Referent		Referent	
Rural	1.24	0.84–1.83	2.92^a^	1.48–5.76	1.01	0.76–1.36	0.89	0.63–1.24
**Age**								
50+	Referent							
30 £ 50	1.46	0.81–2.65	1.78	0.82–3.87	1.05	0.70–1.57	0.88	0.56–1.38
18 < 30	2.49^a^	1.38–4.47	1.72	0.77–3.85	0.92	0.61–1.37	0.94	0.59–1.51
**Relationship status**								
Married/living with a partner	Referent							
Single	1.64^a^	1.09–2.47	1.26	0.77–2.06	1.32	0.78–2.24	1.32	0.76–2.27
Widowed/separated/divorced	0.40	0.12–1.27	0.59	0.17–2.02	0.94	0.66–1.33	0.82	0.55–1.23
**Highest educational level**								
Secondary complete or more	Referent							
Primary complete to secondary incomplete	1.24	0.80–1.92	1.27	0.78–2.09	1.28	0.77–2.14	1.32	0.80–2.18
No schooling to primary incomplete	0.84	0.47–1.52	0.91	0.40–2.09	1.37	0.86–2.19	1.34	0.76–2.38
**Paid work**								
Yes	Referent							
No	1.21	0.78–1.88	1.00	0.56–1.76	1.00	0.74–1.35	1.06	0.78–1.47
**Asset index**								
Upper tercile	Referent							
Middle tercile	0.53^a^	0.31–0.91	0.27^a^	0.13–0.55	1.06	0.76–1.48	0.88	0.58–1.34
Lower tercile	0.98	0.64–1.51	0.37^a^	0.17–0.79	0.94	0.64–1.38	0.80	0.50–1.28
**Breast cancer risk factor knowledge score**	0.88^a^	0.83–0.95	0.91^a^	0.83–0.99	0.98	0.93–1.03	1.07^a^	1.01–1.13
**Breast cancer symptom knowledge score**	0.94^a^	0.90–0.99	0.98	0.90–1.07	0.95^a^	0.91–0.98	0.97	0.92–1.02
**Breast cancer risk factor lay beliefs endorsed**	0.85^a^	0.75–0.97	1.00	0.83–1.20	0.89^a^	0.81–0.98	0.87^a^	0.78–0.97
**Barriers experienced**	1.26^a^	1.18–1.35	1.18^a^	1.08–1.29	1.30^a^	1.25–1.36	1.33^a^	1.26–1.40
**Confident would notice change in breast/s**								
Yes	Referent							
No	2.20^a^	1.38–3.50	1.42	0.78–2.61	1.87^a^	1.35–2.60	1.14	0.78–1.65

a *p* < 0.05

**Table 5.** Factors associated with longer anticipated time to seek care for possible symptoms of cervical cancer.

	SA				Uganda			
	**Prevalence ratio (PR)**	**95% CI PR**	**Adj PR**	**95% CI Adj PR**	**PR**	**95% CI PR**	**Adj PR**	**95% CI Adj PR**
**Location**								
Urban	Referent		Referent		Referent		Referent	
Rural	1.17	0.77–1.77	2.42^a^	1.08–5.45	1.40^a^	1.01–1.94	1.23	0.84–1.78
**Age**								
50+	Referent							
30 £ 50	0.88	0.51–1.51	0.79	0.43–1.43	0.76	0.50–1.16	0.79	0.49–1.27
18 < 30	1.32	0.76–2.27	0.74	0.36–1.52	0.67	0.45–1.12	0.78	0.47–1.29
**Relationship status**								
Married/living with a partner	Referent							
Single	1.55^a^	1.00–2.39	1.26	0.74–2.13	1.14	0.59–2.24	1.37	0.74–2.52
Widowed/separated/divorced	0.73	0.29–1.83	0.64	0.22–1.87	1.32	0.93–1.88	1.04	0.67–1.61
**Highest educational level**								
Secondary complete or more	Referent							
Primary complete to secondary incomplete	1.49	0.92–2.39	1.48	0.85–2.58	1.37	0.70–2.66	1.24	0.64–2.40
No schooling to primary incomplete	1.05	0.56–1.96	1.09	0.47–2.53	2.16^a^	1.19–3.93	1.73	0.85–3.53
**Paid work**								
Yes	Referent							
No	0.90	0.58–1.39	0.62	0.36–1.06	0.92	0.66–1.29	0.84	0.60–1.19
**Asset index**								
Upper tercile	Referent							
Middle tercile	0.60	0.34 –1.06	0.34^a^	0.15–0.80	1.30	0.88–1.91	0.92	0.59–1.44
Lower tercile	1.15	0.73–1.81	0.64	0.27–1.55	1.30	0.85–1.97	0.82	0.50–1.36
**Cervical cancer risk factor knowledge score**	0.95	0.87–1.03	0.97	0.87–1.08	0.91^a^	0.86–0.97	0.94	0.87–1.01
**Cervical cancer symptom knowledge**	0.94	0.88–1.01	0.97	0.86–1.10	0.90^a^	0.86–0.95	0.96	0.90–1.03
**Cervical cancer risk factor lay beliefs endorsed**	1.02	0.80–1.29	1.21	0.93–1.56	1.03^a^	0.85–1.23	1.19	0.96–1.47
**Barriers experienced**	1.23^a^	1.13–1.34	1.19^a^	1.06–1.34	1.28^a^	1.22–1.34	1.23^a^	1.16–1.30
**Confident would notice a symptom that could be cervical cancer**								
Yes								
No	1.60^a^	1.02–2.51	1.28	0.75–2.18	1.76^a^	1.26–2.46	1.24	0.86–1.80

a *p* < 0.05
